# Persistent progression independent of relapse activity in multiple sclerosis

**DOI:** 10.1093/braincomms/fcaf306

**Published:** 2025-08-21

**Authors:** Chao Zhu, Zhen Zhou, Tomas Kalincik, Izanne Roos, Katherine Buzzard, Olga Skibina, Raed Alroughani, Jens Kuhle, Marc Girard, Pierre Grammond, Jeannette Lechner-Scott, Oliver Gerlach, Nevin John, Pamela McCombe, Richard Macdonell, Vincent van Pesch, Guy Laureys, Julie Prevost, Dana Horakova, Eva Kubala Havrdova, Tamara Castillo-Triviño, Cristina Ramo-Tello, Yolanda Blanco, Jose E Meca-Lallana, Alessandra Lugaresi, Valentina Tomassini, Elisabetta Cartechini, Maria Pia Amato, Daniele Spitaleri, Francesco Patti, Davide Maimone, Matteo Foschi, Andrea Surcinelli, Emanuele D'Amico, Bassem Yamout, Samia J Khoury, Maria Jose Sa, Cavit Boz, Serkan Ozakbas, Bianca Weinstock-Guttman, Daniel Merlo, Mastura Monif, Vilija G Jokubaitis, Anneke van der Walt, Helmut Butzkueven

**Affiliations:** Department of Neuroscience, School of Translational Medicine, Monash University, Melbourne, VIC 3004, Australia; School of Public Health and Preventive Medicine, Monash University, Melbourne, VIC 3004, Australia; CORe, Department of Medicine, University of Melbourne, Melbourne, VIC 3000, Australia; Neuroimmunology Centre, Department of Neurology, Royal Melbourne Hospital, Melbourne, VIC 3000, Australia; CORe, Department of Medicine, University of Melbourne, Melbourne, VIC 3000, Australia; Neuroimmunology Centre, Department of Neurology, Royal Melbourne Hospital, Melbourne, VIC 3000, Australia; Eastern Health Clinical School, Monash University, Box Hill, VIC 3128, Australia; Department of Neurology, Box Hill Hospital, Box Hill, VIC 3128, Australia; Eastern Health Clinical School, Monash University, Box Hill, VIC 3128, Australia; Department of Neurology, Box Hill Hospital, Box Hill, VIC 3128, Australia; Department of Neurology, Alfred Hospital, Melbourne, VIC 3004, Australia; Amiri Hospital, Sharq 73767, Kuwait; University Hospital and University of Basel, Basel 4000, Switzerland; CHUM and Universite de Montreal, Montreal, Canada, H2L 4M1; CISSS Chaudière-Appalache, Levis, Canada, G6X 0A1; University Newcastle, Newcastle 2035, Australia; Hunter New England Health, New Lambton, NSW 2050, Australia; Zuyderland Medical Center, Sittard-Geleen 5500, Netherlands; School for Mental Health and Neuroscience, Department of Neurology, Maastricht University Medical Center, Maastricht 6131, Netherlands; School of Clinical Sciences, Monash University, Clayton, VIC 3168, Australia; Department of Neurology, Royal Brisbane Hospital, Brisbane 4000, Australia; Austin Health, Melbourne 3084, Australia; Department of Neurology, Cliniques Universitaires Saint-Luc, Brussels 1200, Belgium; Department of Neurology, University Hospital Ghent, Ghent 9000, Belgium; CSSS Saint-Jérôme, Saint-Jerome, Canada, J7Z 5T3; Department of Neurology and Center of Clinical Neuroscience, Charles University in Prague and General University Hospital, Prague 12808, Czech Republic; Department of Neurology and Center of Clinical Neuroscience, Charles University in Prague and General University Hospital, Prague 12808, Czech Republic; Hospital Universitario Donostia and IIS Biodonostia, San Sebastián 20014, Spain; Department of Neuroscience, Hospital Germans Trias i Pujol, Badalona 8916, Spain; Center of Neuroimmunology, Service of Neurology, Hospital Clinic de Barcelona, Barcelona 8916, Spain; Multiple Sclerosis CSUR and Clinical Neuroimmunology Unit, Neurology Department, Virgen de la Arrixaca Clinical University Hospital, IMIB-Arrixaca, Murcia 30120, Spain; Dipartimento di Scienze Biomediche e Neuromotorie, Università di Bologna, Bologna 40139, Italy; IRCCS Istituto delle Scienze Neurologiche di Bologna, Bologna 40139, Italy; Institute for Advanced Biomedical Technologies (ITAB), Department of Neurosciences, Imaging and Clinical Sciences, University G. d'Annunzio of Chieti-Pescara, Chieti 66013, Italy; Neurology Unit, AST Macerata, Macerata 62100, Italy; Department Neurofarba, University of Florence, Florence 50123, Italy; IRCCS Fondazione Don Carlo Gnocchi, Florence 50143, Italy; Azienda Ospedaliera di Rilievo Nazionale San Giuseppe Moscati Avellino, Avellino 83100, Italy; Department of Medical and Surgical Sciences and Advanced Technologies, GF Ingrassia, Catania 95123, Italy; Azienda Opsedaliera per l'Emergenza Cannizzaro, Catania 95126, Italy; Department of Neuroscience, S. Maria delle Croci Hospital, AUSL Romagna, Ravenna 48121, Italy; Department of Neuroscience, S. Maria delle Croci Hospital, AUSL Romagna, Ravenna 48121, Italy; Medical and Surgical Sciences, Universita di Foggia, Foggia 71122, Italy; Neurology Institute, Harley Street Medical Center, Abu Dhabi 00000, UAE; Nehme and Therese Tohme Multiple Sclerosis Center, American University of Beirut Medical Center, Beirut 1107 2020, Lebanon; Department of Neurology, Centro Hospitalar Universitario de Sao Joao, Porto 4200-319, Portugal; Department of Neurology, Karadeniz Technical University, Medical Faculty, Trabzon 61080, Turkey; Izmir University of Economics, Medical Point Hospital, Izmir 35575, Turkey; Department of Neurology, Jacobs MS Center for Treatment and Research 14203, United States; Department of Neuroscience, School of Translational Medicine, Monash University, Melbourne, VIC 3004, Australia; Department of Neuroscience, School of Translational Medicine, Monash University, Melbourne, VIC 3004, Australia; Department of Neurology, Alfred Hospital, Melbourne, VIC 3004, Australia; Department of Neuroscience, School of Translational Medicine, Monash University, Melbourne, VIC 3004, Australia; Department of Neurology, Alfred Hospital, Melbourne, VIC 3004, Australia; Department of Neuroscience, School of Translational Medicine, Monash University, Melbourne, VIC 3004, Australia; Department of Neurology, Alfred Hospital, Melbourne, VIC 3004, Australia; Department of Neuroscience, School of Translational Medicine, Monash University, Melbourne, VIC 3004, Australia; Department of Neurology, Alfred Hospital, Melbourne, VIC 3004, Australia

**Keywords:** progression independent of relapse activity (PIRA), secondary progressive MS (SPMS), relapsing-remitting multiple sclerosis (RRMS), disability improvement, disability progression

## Abstract

Patients with relapsing-remitting multiple sclerosis (RRMS) may experience disability progression independent of relapse activity (PIRA), which can be an early sign of secondary progressive MS (SPMS). We defined persistent PIRA as ongoing sustained disability over the entire available follow-up period. However, PIRA events can regress over time. Identifying factors that predict PIRA persistence is of great interest as they can refine the definition of RRMS to SPMS transition. Equally, factors associated with the non-persistence of PIRA have potential treatment implications for patients suffering from a PIRA event. We conducted a study to examine risk factors for PIRA persistence and risk differences in long-term disability progression between persistent and non-persistent PIRA. In this cohort study, we included only patients who had already experienced a PIRA event and investigated the persistence of disability progression following their first PIRA event. Therefore, PIRA occurrence time was set as the baseline. Data were collected from the MSBase registry between April 1995 and January 2024, with a median follow-up of 8.7 years. The primary outcome was time to 6-month confirmed non-persistence of PIRA. Secondary outcomes comprised time to 6-month confirmed Expanded Disability Status Scale (EDSS) 6 and time to SPMS. A stratified Cox regression model was used to identify risk factors associated with non-persistent PIRA. We then matched persistent PIRA patients with non-persistent PIRA patients in a 1:1 ratio using propensity scores, and compared their risk of reaching EDSS 6 using the Cox regression model. We re-matched patients with complete Kurtzke Functional Systems Scores to compare their risks of reaching SPMS. We included 4713 RRMS patients with PIRA, of whom around one-third experienced a post-PIRA disability improvement, over a relatively long period (median of 2.6 years to improvement). Use of high-efficacy disease-modifying therapies (DMT) at baseline [hazard ratio, 1.22; 95% confidence interval, (1.08–1.38); *P* = 0.0015], lower baseline EDSS [hazard ratio, 0.73 (0.69–0.78); *P* < 0.0001] and younger age [per 10 years; hazard ratio, 0.84 (0.80–0.89); *P* < 0.0001] were associated with non-persistent PIRA. Patients with non-persistent PIRA had a hazard ratio of 0.19 [95% confidence interval, (0.15–0.25); *P* < 0.0001] for reaching EDSS 6 and 0.18 [(0.11–0.29); *P* < 0.0001] for reaching SPMS compared to patients with persistent PIRA. PIRA events slowly regress in one-third of patients. Patients with persistent PIRA had a substantially higher risk of reaching EDSS 6 and SPMS than those with non-persistent PIRA. Younger age, lower baseline EDSS, and use of high-efficacy DMT during PIRA events were associated with PIRA regression.

## Teaser

Zhu *et al.* reported that patients with persistent progression independent of relapse activity had a higher risk of disability and transition to secondary progressive multiple sclerosis. However, some showed improvement over time. Younger age, lower baseline disability, and high-efficacy treatment were associated with reversal, highlighting the need for nuanced interpretation of progression.

## Graphical Abstract

**Graphical Abstract:**
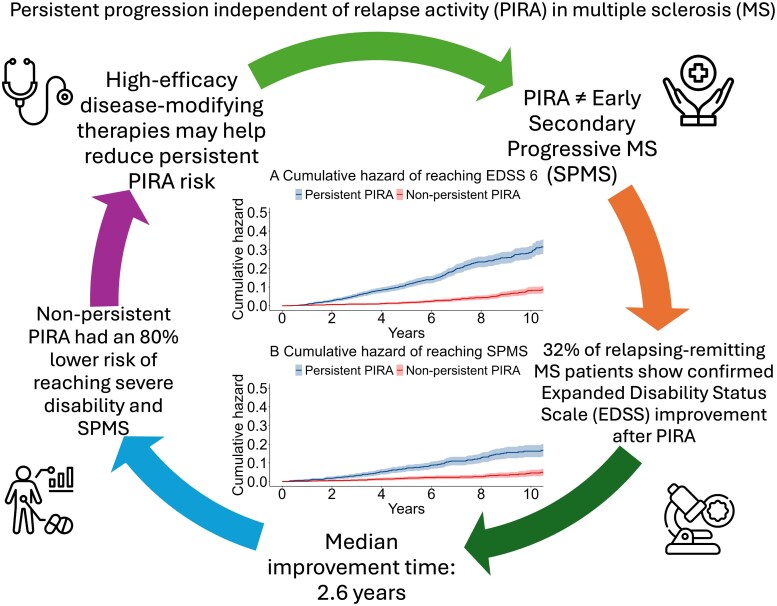

## Introduction

Approximately 85% of individuals with multiple sclerosis (MS) have an initial relapsing-remitting (RRMS) disease course. Relapse-associated neurological disability can leave residual long-term or permanent disability known as relapse-associated worsening (RAW).^1^ However, disability progression can also occur in the absence of relapses, termed progression independent of relapse activity (PIRA).^2-6^ Some PIRA events are an early marker of the transformation from RRMS into secondary progressive MS (SPMS), which is characterized by gradual and continuous disability progression.^7^ People with MS who experience PIRA are known to have accelerated brain volume reduction, primarily attributable to grey matter loss in the cerebral cortex^4^; and greater risk of reaching Extended Disability Status Scale (EDSS) 6.0, a rate that is 8-fold higher than patients without PIRA.^5^ Interestingly, previous studies also found that using high-efficacy disease-modifying therapies (DMTs) is associated with a lower risk of PIRA, suggesting that ongoing inflammation is responsible for at least some PIRA events, not just RAW events.^2,8,9^ Two systematic reviews examining the existing evidence of PIRA have proposed clinically applicable standard definitions of PIRA to direct ongoing research in this area.^10,11^

There has been little study to-date on post-PIRA disability trajectories; specifically, whether patients can improve post-PIRA, and what factors are associated with a higher probability of PIRA regression. It is also unknown whether the long-term risk of SPMS or ongoing disability progression is different after PIRA for patients who experience PIRA regression/non-persistence versus those who do not.

We performed a retrospective longitudinal cohort study using the MSBase registry dataset^12^ to determine the rate of improvement post-PIRA events, assess factors associated with persistent versus non-persistent PIRA, and the long-term outcomes in both groups of patients. Outcomes of interest included time to non-persistent PIRA, confirmed disability progression to EDSS 6, and SPMS.

## Materials and methods

Patient data spanning from April 1995 to January 2024 was extracted from the MSBase registry, an international observational cohort study of MS.^12^ Ethical approval for the MSBase registry was granted by the Alfred Health Human Research and Ethics Committee and the local ethics committees in participating centres. All enrolled patients provided written or verbal consent according to local regulations. Our study followed the Strengthening the Reporting of Observational Studies in Epidemiology (STROBE) reporting guideline.

### Study design and participants

Combining the recommendations of the two systematic reviews,^10,11^ we defined PIRA as a 6-month confirmed disability progression with no relapse for the entire period covering the reference EDSS score (the most recent pre-worsening EDSS), the EDSS score with progression (worsening), and a confirmation EDSS at least 6 months after the EDSS worsening. A 30-day relapse-free period prior to the reference EDSS score was also required to reduce the risk of recent disease activity influencing the reference EDSS score, ensuring it accurately reflects the patient's disability status. A confirmed progression event was defined as an increase in EDSS of ≥1.5, ≥ 1.0 or ≥0.5 from a baseline EDSS of 0, 1.0–5.5 or ≥6.0, respectively. To confirm progression, the increased EDSS had to be sustained for ≥6 months. We defined early PIRA as PIRA occurring within 5 years from the first symptoms of MS,^6^ and early DMT as the first use of DMT within 3 years of MS onset.^11^

Patient data were recorded during routine clinic visits at participating centres via the locally installed iMed or MDS MSBase data entry systems. Patients were included if they were diagnosed as RRMS according to the McDonald criteria,^13^ had at least one PIRA event, and had an EDSS of no more than four at baseline. The baseline was set at the time of the first PIRA occurrence. Participants were also required to have at least three visits with documented EDSS during the follow-up to allow for the ascertainment of disability accumulation and identification of the occurrence of PIRA, and at least three more EDSS visits after the PIRA event to assess disability improvement and/or PIRA persistence. Patients were excluded from the study if they had been diagnosed with PPMS and SPMS before the occurrence of PIRA. In MSBase, a relapse is defined as a new or recurrent neurological symptom lasting for 24 h or more without fever or infection.^14^

### Study outcomes and definitions

The primary study outcome was time to 6-month confirmed non-persistent PIRA, defined as an improvement in EDSS of ≥1.0 or ≥0.5 from the PIRA occurrence of 2.0–5.5 or ≥6.0, respectively, again with a confirmation period of ≥6 months. Absence of relapses was also required between the 30 days before EDSS improvement and the confirmation period. Conversely, persistent PIRA was defined as an ongoing sustained or worsening EDSS disability score that did not improve to the end of follow-up after the PIRA occurrence.

The secondary outcomes were time to 6-month confirmed EDSS 6.0 and time to SPMS. We defined SPMS according to the criteria of Lorscheider *et al*.,^15^ as a disability progression in EDSS by 1.0 step if the reference EDSS score (the most recent EDSS assessment before SPMS conversion) was ≤5.5, or 0.5 step if the reference EDSS score was ≥6.0 before the SPMS conversion, leading to an EDSS of at least 4.0, and was confirmed with a ≥3-month period, with a pyramidal functional score of ≥2. We did not allow a relapse between the reference EDSS assessment and SPMS conversion. In the unlikely event that the interval between the reference EDSS and the SPMS conversion was less than 30 days, no relapse was allowed within the 30 days preceding the SPMS conversion to ensure the progression was not relapse-related. This secondary outcome of time to SPMS could only be analyzed in patients with complete Kurtzke Functional Systems Scores (KFS, a scoring system for assessing neurological impairment in MS that includes visual function, brainstem function, pyramidal function, cerebellar function, sensory function, bowel/bladder function and mental function).

### Statistical analysis

Continuous variables with a normal distribution were expressed as mean (standard deviation), otherwise as median (interquartile), and categorical variables were expressed as frequency (percentage). Risk factors associated with non-persistent PIRA were first assessed in univariate analyses using Cox proportional-hazards models. Age per 10 years, sex, early PIRA, high-efficacy DMT use within 3 months before baseline, the number of DMTs used before baseline, relapses in one and two years before baseline, baseline EDSS, disease duration, early DMT, and the proportion of DMT use period were examined as potential risk factors in univariate models. All variables with *P* < 0.20 in univariate analyses or those of known clinical relevance were included in multivariable analyses. Treatment exposures were adjusted as a time-varying variable in all Cox proportional-hazards models. Due to the violation of the proportional-hazards assumption for time-varying treatment exposures in the multivariable model, we split the entire follow-up period into 3 time intervals, and then used the stratified Cox proportional-hazards model taking into account the interaction of the stratified time intervals with treatment exposures.^16,17^ The proportional-hazards assumption was checked by the Schoenfeld global test.^18^ The variance inflation factor was calculated to test for multicollinearity. After variable selection, a final model was constructed using a backward-stepwise method including only significant variables.

We used propensity score matching to minimize indication bias due to the baseline differences between persistent and non-persistent PIRA patients. A logistic regression model was used to estimate individual propensity scores. Exposure to non-persistent PIRA was the dependent variable, and the risk factors obtained in the previous step and the clinically important variables were the independent variables, including age, disease duration, sex, baseline EDSS, high-efficacy DMT use at baseline, number of DMTs used before baseline, and relapses in two years before baseline. We matched patients based on the corresponding propensity scores using nearest-neighbour matching without replacement with a variable matching ratio of 1:1. We assessed the covariate balance using an absolute standardized difference (ASD), with an ASD > 0.1 indicating an imbalance.^19^ Hazard ratios (HRs) of reaching EDSS 6.0 and SPMS were estimated using a Cox proportional-hazards model with robust standard errors based on matched data. Kaplan–Meier cumulative hazard curves were used to show the cumulative risk for each outcome between persistent and non-persistent PIRA groups. We also performed a subgroup analysis by early PIRA and early DMT as well as variables used for computing propensity scores for the analyses of two secondary outcomes.

All statistical tests were two-sided with significance defined as *P* < 0.05. All analyses were performed in R, version 4.2.2 (R Foundation for Statistical Computing). Data were analysed from 2 January 2024 to 23 March 2024.

### Sensitivity analysis

The missing rate of KFS scores was high (46% of patients were completely or partially missing KFS scores) in the primary analysis. However, previous studies showed that some KFS scores such as bowel/bladder, sensory and pyramidal function, were associated with the occurrence of PIRA.^6,11^ We, therefore, tested the association of all seven functional scores with the persistent PIRA in a sensitivity analysis. We defined a baseline binary variable to indicate whether the PIRA events were driven by an increase in the corresponding functional scores, and repeated the same process as in primary analyses to test for risk factors. We also repeated the time to SPMS analyses using clinician-defined SPMS rather than the Lorscheider criteria to examine the consistency of the results.

## Results

**Figure 1:**
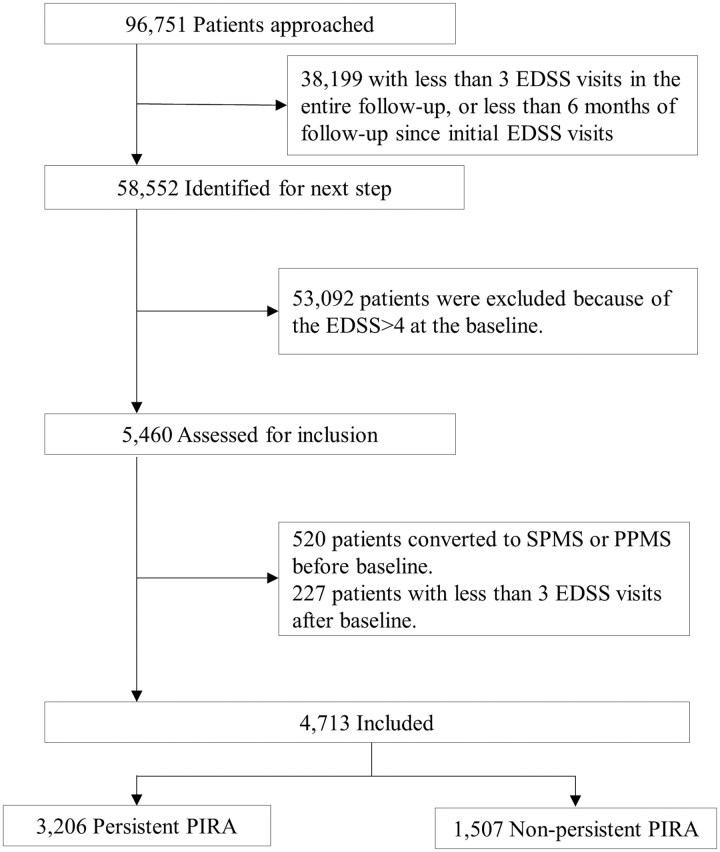
**Flow chart of the patients inclusion/exclusion.** EDSS, Expanded Disability Status Scale; PIRA, Progression Independent of Relapse Activity; MS, Multiple Sclerosis; SPMS, Secondary Progressive MS; PPMS, Primary Progressive MS.

After applying the inclusion criteria, 4713 of 96 751 RRMS patients from 29 countries and 88 centres (outcomes recorded between April 1995 and January 2024) were included in the analysis (Fig. 1, Table 1), with a median follow-up time of 8.7 [(IQR), 6.7–12.0] years. Of these, 3206/4713 (68%) of patients showed no improvement in disability until the end of follow-up after PIRA, and 1507/4713 (32%) of patients improved after PIRA with a median improvement time of 2.6 (IQR, 1.7–4.3) years (Supplementary Fig. 1). Patients with non-persistent PIRA were younger than those with persistent PIRA [mean: 40.99 years (SD 10.03) versus 44.02 years (SD 10.02)], and were 5% more likely to be exposed to high-efficacy DMTs during the PIRA event than patients with persistent PIRA. Patients with persistent PIRA had a worse disability, a longer disease duration, delayed use of initial DMT, and a higher proportion of PIRA occurring within 5 years after symptom onset than patients with non-persistent PIRA.

**Table 1:** Characteristics of the study population by PIRA persistence, and risk factors for PIRA regression in univariate and multivariable analyses

				Univariate^a^		Multivariable^a^	
Risk factors	Total (*n* = 4713)	Persistent PIRA (*n* = 3206)	Non-persistent PIRA (*n* = 1507)	HR (95% CI)	*P*-value	HR (95% CI)	*P*-value
Age, per 10 years	4.31 ± 1.01	4.4 ± 1.00	4.10 ± 1.00	0.81 (0.76–0.85)	<0.0001	0.84 (0.80–0.89)	<0.0001
Sex				0.90 (0.80–1.01)	0.0628		
Male	1282 (27)	894 (28)	388 (26)				
Female	3431 (73)	2312 (72)	1119 (74)				
High-efficacy DMT use during PIRA^b^	843 (18)	525 (16)	318 (21)	1.20 (1.06–1.36)	0.0034	1.22 (1.08–1.38)	0.0015
The number of DMTs used before the baseline^c^	0.0 (0.0–1.0)	0.0 (0.0–1.0)	0.0 (0.0–1.0)	1.02 (0.97–1.08)	0.4060		
The number of relapses in 1 year before baseline	0.0 (0.0–0.0)	0.0 (0.0–0.0)	0.0 (0.0–0.0)	1.02 (0.91–1.14)	0.7609		
The number of relapses in 2 years before baseline	0.0 (0.0–1.0)	0.0 (0.0–1.0)	0.0 (0.0–1.0)	1.01 (0.95–1.07)	0.7314		
Disability (baseline EDSS)	2.5 (2.0–3.5)	3.0 (2.0–3.5)	2.0 (2.0–3.0)	0.71 (0.67–0.76)	<0.0001	0.73 (0.69–0.78)	<0.0001
Disease duration, years	9.4 (5.1–15.2)	10.0 (5.4–15.8)	8.3 (4.4–13.8)	0.98 (0.97–0.99)	<0.0001		
The proportion of DMT use period	0.4 (0.1–0.7)	0.4 (0.0–0.7)	0.4 (0.1–0.8)	1.08 (0.92–1.27)	0.3241		
Early DMT	1888 (40)	1193 (37)	695 (46)	1.21 (1.09–1.34)	0.0003		
Early PIRA	1151 (24)	724 (23)	427 (28)	1.23 (1.10–1.37)	0.0003		

Values are median (interquartile range), *n* (%), or mean ± SD.

^a^Variables with *P* < 0.20 in univariate analyses or those of known clinical relevance were included in multivariable analyses, and only the variables included in the final model were reported in the multivariable columns. Treatment exposures were adjusted as a time-varying variable in all Cox proportional-hazards models.

^b^High-efficacy DMTs include natalizumab, rituximab, ocrelizumab, ofatumumab, stem cell transplant, alemtuzumab, mitoxantrone, fingolimod and cladribine.

^c^The baseline was set at the time of PIRA occurrence.

PIRA, progression independent of relapse activity; DMT, disease-modifying therapies; EDSS, expanded disability status scale.

### Risk factors for persistent and non-persistent PIRA

To determine risk factors for non-persistent PIRA, we constructed a multivariable model as described above. The final multivariable model revealed that age [per 10 years; HR (95% CI), 0.84 (0.80–0.89); *P* < 0.0001], high-efficacy DMTs use within 3 months before baseline [1.22 (1.08–1.38); *P* = 0.0015], and baseline EDSS [0.73 (0.69–0.78); *P* < 0.0001] were associated with non-persistent PIRA events. The detailed results are reported in Table 1. Interactions between all variables were tested and were found to be non-significant.

### Risk of reaching EDSS 6 and SPMS

**Figure 2:**
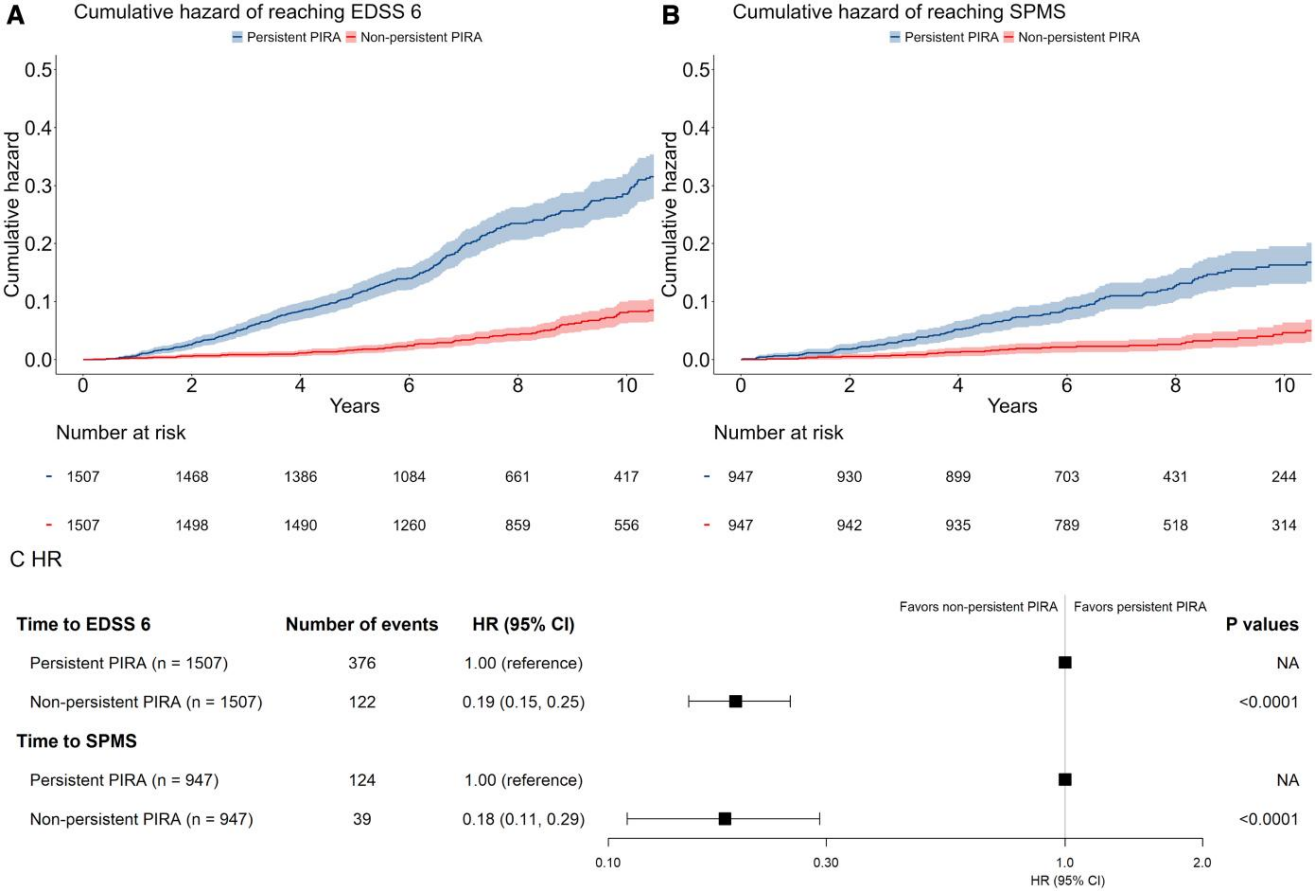
**The cumulative hazard of the secondary outcomes.** Kaplan–Meier failure function was applied to the present cumulative hazard of the: **A** time to EDSS 6 in patients with non-persistent PIRA and patients with persistent PIRA, and **B** time to SPMS, respectively. for **C** hazard ratios for two secondary outcomes. Statistical significance was assessed using the Wald test, with a *P*-value < 0.05 considered significant. CI, confidence interval; HR, hazard ratio; SPMS, secondary progressive multiple sclerosis; EDSS, expanded disability status scale; PIRA, progression independent of relapse activity.

Patients included in the primary analyses were divided into two groups—either persistent or non-persistent PIRA, and then were matched according to their propensity scores. In total, 3014 patients (1507 in each group) were included in the time to 6-month confirmed EDSS 6.0 analysis. Patients with non-persistent PIRA had a significantly 81% lower risk of reaching EDSS 6.0 [HR 0.19; 95% CI, (0.15–0.25); *P* < 0.0001] compared with patients in the persistent PIRA group (Fig. 2).

We rematched only the patients with reported Kurtzke functional pyramidal scores to estimate the differences in risk of reaching SPMS between persistent and non-persistent PIRA groups, as pyramidal scores are required as part of the Lorscheider definition of SPMS. We excluded the patients who had already met the Lorscheider criteria with an EDSS = 4 and pyramidal functional score ≥2 at baseline. Among the patients with a complete pyramidal functional score, 313/2687 (12%) reached SPMS with a median follow-up of 8.0 years. One thousand eight hundred and ninety-four patients (947 in each group) were matched for time to SPMS analysis. Patients with non-persistent PIRA had a significantly lower risk of reaching SPMS compared to patients with persistent PIRA, with an HR of 0.18 [95% CI, (0.11–0.29); *P* < 0.0001]. The Kaplan–Meier cumulative hazard curves and the results for both time to EDSS 6 and SPMS are presented in Fig. 2, and the balanced characteristics of patients are reported in Table 2.

**Table 2:** Baseline characteristics by PIRA persistence for secondary analyses, matched using propensity scores

Matched patients for EDSS 6					
	Total	Persistent PIRA	Non-persistent PIRA	ASD^a^	
				Before matching	After matching
*n*	3014	1507	1507		
Age, years	41.06 ± 9.71	41.13 ± 9.38	40.99 ± 10.03	**0**.**30**	0.01
Sex				0.05	0.02
Male	765 (25)	377 (25)	388 (26)		
Female	2249 (75)	1130 (75)	1119 (74)		
High-efficacy DMT use during PIRA	640 (21)	322 (21)	318 (21)	**0**.**12**	0.01
The number of DMTs used before the baseline	0.0 (0.0–1.0)	0.0 (0.0–1.0)	0.0 (0.0–1.0)	0.03	0.02
The number of relapses in 2 years before baseline	0.0 (0.0–1.0)	0.0 (0.0–1.0)	0.0 (0.0–1.0)	0.06	0.01
Disability (baseline EDSS)	2.0 (2.0–3.0)	2.0 (2.0–3.0)	2.0 (2.0–3.0)	**0**.**40**	0.01
Disease duration, years	8.3 (4.5–13.7)	8.4 (4.6–13.4)	8.3 (4.4–13.8)	**0**.**21**	0.01

Matched patients for SPMS					
*n*	1894	947	947		
Age, years	40.89 (9.90)	41.15 (9.15)	40.63 (10.16)	**0**.**31**	0.05
Sex				0.07	0.02
Male	489 (26)	248 (26)	241 (25)		
Female	1405 (74)	699 (74)	706 (75)		
High-efficacy DMT use	391 (21)	188 (20)	203 (21)	0.08	0.04
The number of DMTs use	0.0 (0.0–1.0)	0.0 (0.0–1.0)	0.0 (0.0–1.0)	0.03	0.03
The number of relapses in 2 years before baseline	0.0 (0.0–1.0)	0.0 (0.0–1.0)	0.0 (0.0–1.0)	0.04	0.02
Disability (baseline EDSS)	2.0 (2.0–3.0)	2.0 (2.0–3.0)	2.0 (2.0–3.0)	**0**.**42**	<0.01
Disease duration, years	8.0 (4.5–13.0)	7.9 (4.6–13.0)	8.1 (4.5–13.3)	**0**.**21**	0.02

Values are median (interquartile range), *n* (%), or mean ± SD.

^a^ASD is the absolute difference in means or proportions divided by the standard error. An imbalance was defined as an ASD > 0.10, indicated in bold.

ASD, absolute standardized difference; PIRA, progression independent of relapse activity; DMT, disease-modifying therapies; EDSS, expanded disability status scale; SPMS, secondary progressive multiple sclerosis.

Results in the defined subgroups were generally consistent with the results in the main analysis. Non-persistent PIRA significantly interacted with high-efficacy DMT use at baseline and early DMT use on the risk of reaching EDSS 6 (*P* < 0.05 for interaction), and the associations were greater for patients treated with high-efficacy DMTs at baseline and those treated earlier (Supplementary Table 1). No significant interactions were detected between the subgroups and non-persistent PIRA for the risk of reaching SPMS (Supplementary Table 2).

### Sensitivity analyses

The primary analyses for risk factors were repeated with all potential risk factors and the seven functional scores in patients with a complete KFS score. Age per 10 years, high-efficacy DMT use within 3 months before baseline, baseline EDSS, disease duration, early PIRA, early DMT, the proportion of DMT use period, visual function, brainstem function, pyramidal function and sensory function satisfied the criteria in the univariate analyses and were included in the multivariable analysis (Supplementary Table 3). Overall, age [per 10 years; HR (95% CI), 0.82 (0.76–0.90); *P* < 0.0001], baseline EDSS [0.73 (0.67–0.80); *P* < 0.0001], visual function [0.82 (0.71–0.95); *P* = 0.0066], brainstem function [0.75 (0.63–0.89); *P* < 0.0001] and, marginally, pyramidal function [0.82 (0.68–1.00); *P* = 0.0447] were all associated with a lower probability of PIRA regression. Moreover, high-efficacy DMTs use within 3 months before baseline [1.20 (1.02–1.42); *P* = 0.0253] was significantly associated with non-persistent PIRA.

As an additional sensitivity analysis, we used clinician-defined SPMS as the outcome. 266/2687 (10%) of patients were diagnosed with SPMS by their treating clinicians. Compared with persistent PIRA, patients with non-persistent PIRA had a significantly lower risk of reaching clinician-defined SPMS, with an HR of 0.19 (95% CI, 0.04–0.84; *P* = 0.0292).

## Discussion

In this retrospective cohort study, we showed that 32% of RRMS patients experienced 6-month confirmed EDSS improvement after a PIRA event, i.e. a regression of PIRA. This improvement was very slow, with a median time to improvement of 2.6 years. Younger age, high-efficacy DMT use within 3 months before PIRA event and lower baseline EDSS were factors associated with non-persistent PIRA. While PIRA is not necessarily synonymous with early secondary progressive MS, it appears to represent a heterogeneous event. Given the lack of strong biomarkers for secondary progression,^20^ the evolving definitions of secondary progressive MS, and the possibility of an underlying progressive process despite some improvement in PIRA events, further research is needed to fully understand its relationship to SPMS. In cases of non-persistent PIRA, we found more than an 80% lower risk of reaching severe disability and SPMS compared to persistent PIRA.

Many prior studies showed that age was a major risk factor for PIRA.^2,5,21^ Our findings confirmed and extended previous research by showing that increasing age also increased the likelihood of persistent PIRA. Specifically, each additional 10 years of age was associated with a 16% decrease in the likelihood of experiencing non-persistent PIRA. Previously, PIRA was identified as a major contributor to disability accumulation in people of all ages.^22^ Our findings extended this observation that persistent PIRA was associated with a much greater risk of long-term disability progression. Furthermore, prior research also demonstrated that a more severe baseline disability was associated with a higher risk of PIRA.^2,22,23^ Our findings again extended this observation, as higher baseline disability was also a crucial factor associated with greater odds of long-term PIRA persistence.

Previous evidence indicated that higher pyramidal functional scores were associated with greater risk of sustained disability progression,^7,24,25^ which was also shown to be a factor driving persistent PIRA in our study. We also found that PIRA events driven by increased vision and brainstem functional scores were more likely to persist. Brainstem progression potentially reflects progressive dysarthria, bulbar dysfunction and disorders of conjugate gaze and visual worsening independent of acute optic neuritis reflect a progressive optic neuropathy. A previous systematic review on the prognostic impact of brainstem dysfunction did not consistently identify brainstem dysfunction as a risk factor for SPMS.^26^ However, to our knowledge, no prior study had assessed the prognostic significance of sustained worsening of brainstem symptoms. PIRA events driven by worsening visual functional scores are previously believed to be very rare, with relevant literature restricted to case reports.^27,28^ One report described a case of visual PIRA concomitant with other progression features. In our study, visual worsening was detected as the only driver of PIRA in 180/2100 (9%) PIRA events in patients with complete KFS scores. We also found that visual KFS-led PIRA events had a higher rate of long-term persistence. It is therefore likely that subtle progressive visual dysfunction in the absence of acute optic neuritis relapses is more common than previously reported. It is possible that investigators have not paid much attention to slowly worsening vision in middle-aged individuals with MS, as there are, of course, many non-MS-associated causes. Visual and other PIRA events could possibly be driven by slowly expanding lesions (SELs), but dedicated imaging correlation studies are required.^29^

Several divergent perspectives exist regarding the efficacy of DMT in mitigating the risk of PIRA. Whilst some studies failed to demonstrate a favourable impact of DMT use on PIRA,^21,30^ many more showed that the use of DMT can significantly delay the time to reach milestone EDSS values and reduce the risk of experiencing PIRA.^2,7-9,22,31^ Our findings showed, for the first time, that patients who used high-efficacy DMT during PIRA were 22% more likely to experience non-persistent PIRA, i.e. to improve their disability again. We speculate that some PIRA events could have an underlying inflammatory aetiology, so that exposure to high-efficacy therapy during PIRA events increased the probability of ultimate improvement perhaps by limiting the extent of inflammatory injury. Other PIRA events probably reflected an underlying slow neurodegeneration that no current therapy can target, and therefore a greater likelihood of long-term persistence. In the future, a close correlation between clinical and imaging trajectories could clarify if specific imaging correlates of PIRA and post-PIRA improvement exist, for instance slowly expanding lesion dynamics.

Previous findings suggested that patients with PIRA had a higher risk of reaching EDSS 6 than those without,^5,31^ and that PIRA events early in the disease course were also tied to a higher risk of SPMS.^6^ Our results are consistent with these prior observations, but also extended them—PIRA with long-term persistence carried a much higher association with subsequent disability worsening than the one-third of PIRA that ultimately reverted (the reversion rate is consistent with previous findings in RRMS^7^).

Several limitations should be taken into account. Previous results showed that patients with PIRA presented with accelerated brain atrophy, particularly in the cerebral cortex, and a greater loss of brain volume was found in patients with PIRA compared to clinically stable patients.^4,23^ It would, therefore, be worth examining the risk factors and differences in persistent and non-persistent PIRA groups using MRI data, as already discussed above. However, we could not include MRI information in our analysis due to the absence of MRI data in around 65% of study participants. Determining when MS changes from relapsing to progressive phenotype is not easy—it is a gradual process on a continuum. Still, we need a clear definition for research purposes, even if it is simplified.^32^ The Lorscheider criteria can objectively and repeatably determine when secondary progression first appears clinically, making it suitable for registry data studies.^15^ We also considered clinician-defined SPMS, which might be more variably applied but reflects real-world practice. It is reassuring that both approaches yielded similar results in our study. In addition, while our study design aimed to minimise bias by using the first PIRA event as a shared baseline, we acknowledge potential limitations in defining persistent/non-persistent PIRA groups based on future EDSS assessments. This may lead to interference between the group classification and the outcome measurement,^33^ because patients could achieve non-persistence beyond the known/recorded follow-up period and might therefore be incorrectly classified as persistent PIRA. However, any long-term study has to accommodate variable follow-up time, and we still detected sufficient numbers of non-persistent PIRA events to characterise this outcome in detail.

## Conclusion

This study examined the concept of non-persistent PIRA, defined as an eventual improvement in EDSS after a PIRA event. We showed that this occurred in one-third of all observed PIRA events in people with MS enrolled in the MSBase cohort study. Interestingly, the improvement took place over a long period, with a median of more than 2 years. Non-persistent PIRA is more frequent in younger patients and at lower EDSS scores, and more likely in patients using high-efficacy DMT during PIRA events. All of these risk factors suggest an inflammatory aetiology in some PIRA events. Patients with long-term persistent PIRA are at a substantially higher risk of reaching EDSS 6 and SPMS than those with non-persistent PIRA.

## Supplementary Material

fcaf306_Supplementary_Data

## Data Availability

To protect participant confidentiality, de-identified patient-level data sharing may be possible in principle, but will require permissions/consent from each contributing data controller. Analysis R codes are described in the Supplementary material.
