# Supplementary material for: Persistent progression independent of relapse activity in multiple sclerosis
Source: Brain Commun. 2025 Aug 21;7(5):fcaf306. doi: 10.1093/braincomms/fcaf306 (PMC12405762; doi:10.1093/braincomms/fcaf306)

Supplementary Table 1 Subgroup analyses for EDSS 6		
Subgroups	Adjusted HR (95% CI)	P values for interaction
Age, yrs		
< 42·8	0.27 (0.21 – 0.35)	0·3016
⩾42·8	0.23 (0.17 – 0.31)	
Gender		
Female	0.26 (3·12 – 4·90)	0·9837
Male	0.25 (0.18 – 0.36)	
Disease duration, yrs		
< 9·4	0.27 (0.21 – 0.35)	0·5498
⩾9·4	0.24 (0.18 – 0.32)	
Disability (EDSS)		
< 2·5	0.25 (0.19 – 0.34)	0·6031
⩾2·5	0.24 (0.19 – 0.31)	
The number of relapses in two years before baseline		
= 0	0.25 (0.20 – 0.32)	0·7789
> 0	0.26 (0.19 – 0.35)	
The number of DMTs used before the baseline		
< 0	0.27 (0.22 – 0.34)	0·4944
⩾0	0.23 (0.16 – 0.32)	
High-efficacy DMT use during PIRA, yrs		
= 0	0.28 (0.23 – 0.34)	**0·0359**
= 1	0.16 (0.09 – 0.27)	
Early DMT		
= 0	0.30 (0.24 – 0.38)	**0·0329**
= 1	0.19 (0.13 – 0.26)	
Early PIRA		
= 0	0.27 (0.22 – 0.34)	0·2603
= 1	0.21 (0.14 – 0.32)	
The proportion of DMT use period		
<0·37	0.28 (0.22 – 0.36)	0·3545
⩾0·37	0.23 (0.17 – 0.31)	

**Supplementary Table 2 Subgroup analyses for SPMS**		
Subgroups	Adjusted HR (95% CI)	P values for interaction
Age, yrs		
< 42·4	0.28 (0.18 – 0.43)	0·6431
⩾42·4	0.23 (0.13 – 0.42)	
Gender		
Female	0.26 (0.17 – 0.40)	0.6414
Male	0.21 (0.11 – 0.39)	
Disease duration, yrs		
< 8·8	0.20 (0.12 – 0.34)	0.1364
⩾8·8	0.32 (0.20 – 0.50)	
Disability (EDSS)		
< 2·5	0.22 (0.13 – 0.37)	0.5573
⩾2·5	0.28 (0.18 – 0.44)	
The number of relapses in two years before baseline		
= 0	0.27 (0.18 – 0.42)	0.4174
> 0	0.21 (0.12 – 0.36)	
The number of DMTs used before the baseline		
< 0	0.23 (0.15 – 0.35)	0.3221
⩾0	0.31 (0.18 – 0.54)	
High-efficacy DMT use during PIRA, yrs		
= 0	0.28 (0.20 – 0.40)	0.2071
= 1	0.14 (0.05 – 0.40)	
Early DMT		
= 0	0.29 (0.19 – 0.44)	0.3167
= 1	0.20 (0.11 – 0.36)	
Early PIRA		
= 0	0.28 (0.19 – 0.41)	0.4013
= 1	0.20 (0.10 – 0.41)	
The proportion of DMT use period		
<0·38	0.25 (0.15 – 0.41)	0·7514
⩾0·38	0.26 (0.17 – 0.41)	

Supplementary Table 3 Characteristics of the Study Population by PIRA Persistence, and the significant risk factors in the univariate and multivariable analyses for sensitivity analysis 1.

				Univariate		Multivariable	
**Risk factors**	**Total (n=2,100)**	**Persistent PIRA (n=1,365)**	**Non-persistent PIRA (n=735)**	**HR (95% CI)**	***P*-value**	**HR (95% CI)**	***P*-value**
**Age, per 10 yrs**	4.20 (0.99)	4.30 (0.97)	4.00 (0.99)	0·77 (0·72 – 0·84)	<0·0001	0.82 (0.76 – 0.90)	<0·0001
**Sex**				0·92 (0·79 – 1·08)	0·3343	–	–
**Male**	609 (29)	404 (30)	205 (28)			–	–
**Female**	1491 (71)	961 (70)	530 (72)			–	–
**High-efficacy DMT use during PIRA[†]**	552 (26)	348 (25)	204 (28)	1·16 (0·99 – 1·37)	0·0699	1·20 (1·02 – 1·42)	0·0253
**The number of DMTs used before the baseline[‡]**	0·0 (0·0 – 1·0)	0·0 (0·0 – 1·0)	0·0 (0·0 – 1·0)	1·00 (0·92 – 1·08)	0·9168	–	–
**The number of relapses in one year before baseline**	0·0 (0·0 – 0·0)	0·0 (0·0 – 0·0)	0·0 (0·0 – 0·0)	0·94 (0·80 – 1·10)	0·4189	–	–
**The number of relapses in two years before baseline**	0·0 (0·0 – 1·0)	0·0 (0·0 – 1·0)	0·0 (0·0 – 1·0)	1·01 (0·93 – 1·10)	0·7894	–	–
**Disability (baseline EDSS)**	2·5 (2·0 – 3·5)	2·5 (2·0 – 3·5)	2·0 (2·0 – 3·0)	0·69 (0·63 – 0·76)	<0·0001	0·73 (0·67 – 0·80)	<0·0001
**Disease duration, yrs**	8·6 (5·0 – 14·0)	9·1 (5·3 – 14·6)	7·7 (4·3 – 12·6)	0·98 (0·97 – 0·99)	<0·0001	–	–
**the proportion of DMT use period**	0·6 (0·3 – 0·8)	0·5 (0·3 – 0·8)	0·6 (0·3 – 0·8)	1·43 (1·12 – 1·83)	0·0046	–	–
**Early DMT**	1155 (55)	721 (53)	434 (59)	1·25 (1·08 – 1·45)	0·0028	–	–
**Early PIRA**	529 (25)	314 (23)	215 (29)	1·29 (1·10 – 1·51)	0·0019	–	–
**Visual**	1131 (54)	755 (55)	376 (51)	0·88 (0·76 – 1·01)	0·0727	0·82 (0·71 – 0·95)	0·007
**Brainstem**	658 (31)	475 (35)	183 (25)	0·68 (0·57 – 0·80)	<0·0001	0·75 (0·63 – 0·89)	<0·0001
**Pyramidal**	446 (21)	309 (23)	137 (19)	0·82 (0·68 – 0·99)	0·0392	0·82 (0·68 – 1·00)	0·0447
**Cerebellar**	960 (46)	614 (45)	346 (47)	1·07 (0·92 – 1·23)	0·3764	–	–
**Sensory**	545 (26)	377 (28)	168 (23)	0·83 (0·70 – 0·99)	0·0411	–	–
**Bowel/Bladder**	405 (19)	266 (19)	139 (19)	0·97 (0·81 – 1·17)	0·7655	–	–
**Mental**	391 (19)	264 (19)	127 (17)	0·92 (0·76 – 1·11)	0·3753	–	–

Values are median (interquartile range), n (%), or mean ± SD.
[*]Variables with P<0·20 in univariate analyses or those of known clinical relevance were included in multivariable analyses, and only the variables included in the final model were reported in the multivariable columns. Treatment exposures were adjusted as a time-varying variable in all Cox proportional-hazards models.
[†]High-efficacy DMTs include natalizumab, rituximab, ocrelizumab, ofatumumab, stem cell transplant, alemtuzumab, mitoxantrone, fingolimod, and cladribine.
[‡]The baseline was set at the time of PIRA occurrence.
PIRA = Progression Independent Of Relapse Activity; DMT = Disease-Modifying Therapies; EDSS = Expanded Disability Status Scale.

Supplementary Figure 1 Time to non-persistent PIRA

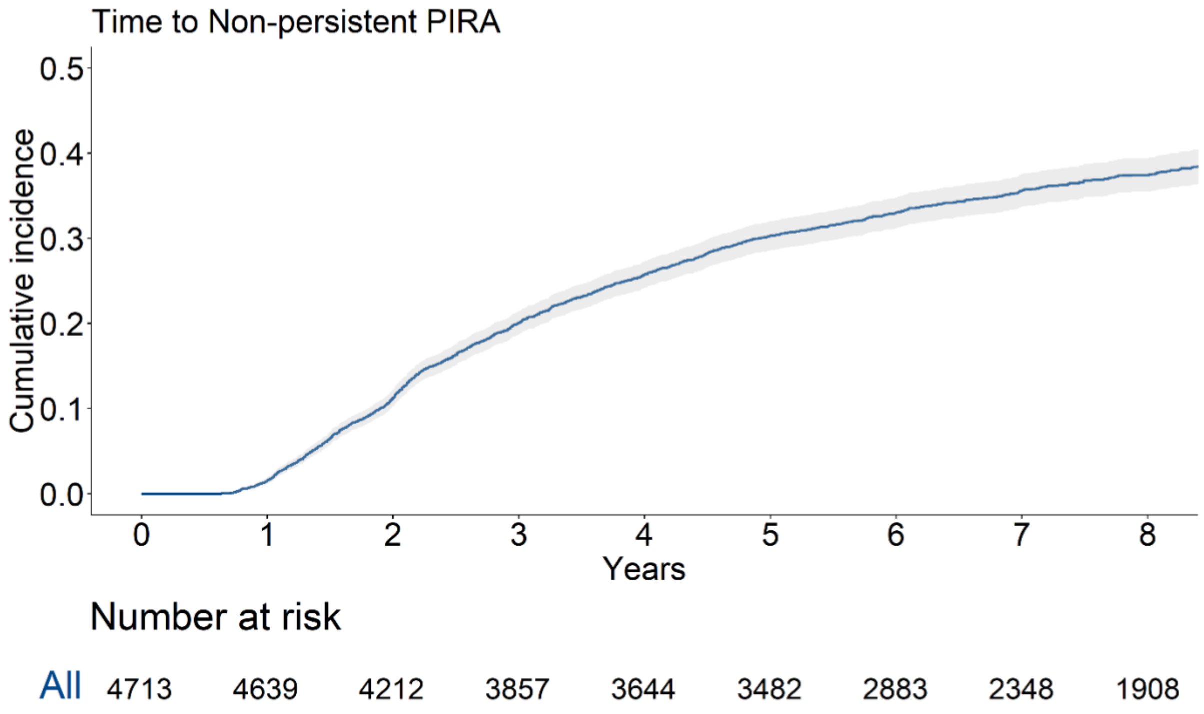

Supplement: fcaf306_Supplementary_Data [file fcaf306_supplementary_data.zip › Supplementary_Materials.pdf]
